# Mini-Invasive floating metatarsal osteotomy for resistant or recurrent neuropathic plantar metatarsal head ulcers

**DOI:** 10.1186/s13018-016-0414-x

**Published:** 2016-07-11

**Authors:** Eran Tamir, Aharon S. Finestone, Erez Avisar, Gabriel Agar

**Affiliations:** Department of Orthopaedic Surgery, Assaf HaRofeh Medical Center, Zerrifin, Affiliated to the Sackler School of Medicine, Tel Aviv University, Tel Aviv, Israel; Maccabi Health Services, POB 1424, Reut, 71799 Israel

**Keywords:** Diabetic foot, Prophylactic surgery, Metatarsal osteotomy, Amputation

## Abstract

**Background:**

Patients with peripheral neuropathy and pressure under a relatively plantar deviated metatarsal head frequently develop plantar foot ulcers. When conservative management with orthotics and shoes does not cure the ulcer, surgical metatarsal osteotomy may be indicated to relieve the pressure and enable the ulcer to heal. The purpose of this study is to evaluate the use of a mini-invasive floating metatarsal osteotomy in treating recalcitrant ulcers or recurrent ulcers plantar to the metatarsal heads in patients with diabetes mellitus (DM) related neuropathy.

**Methods:**

Computerized medical files of patients with diabetic neuropathy treated with an osteotomy during 2013 and 2014 were retrospectively reviewed. There were 20 osteotomies performed on 17 patients (mean age 58 years). The patients had a diagnosis of DM for a mean of 17 years. All ulcers were University of Texas grade 1A; mean ulcer age was 19 months.

**Results:**

After 17/20 operations, the ulcer completely resolved after 6 weeks and did not recur after a mean follow-up of 11.5 months. One patient developed an early post-operative infection with osteomyelitis at the osteotomy site (proximal shaft of the fifth metatarsal) that needed debridement and IV antibiotics. In the other 19 cases, the surgical wound healed within 1 week. Asymptomatic radiological non-union developed in six cases (30 %).

**Conclusions:**

Mini-invasive floating metatarsal osteotomy can cure resistant and recurrent University of Texas grade 1A ulcerations plantar to the metatarsal heads in neuropathic patients.

## Background

Pressure ulcers are common complications in patients with peripheral neuropathy. Most peripheral neuropathy is related to diabetes mellitus (DM). The annual incidence of ulcers in patients with DM is about 2 % [1], and they have been implicated as a causative factor in up to 84 % of diabetic foot amputations [2].

In the presence of sensory neuropathy and lack of protective sensation, an ulcer can develop in a foot with normal anatomy as result of an acute injury. More often, however, an ulcer develops when an anatomic abnormality of the foot is present that results in increased local pressure. A frequent site of ulcers is under the heads of the metatarsals [3], where a flexion deformity (relative to the other metatarsals) results in elevated local pressure [4].

Primary off-loading with a cast is usually an effective treatment to achieve ulcer closure [5–7]. Afterwards, continued care is given with special shoes and orthotics to prevent recurrence. But these conservative methods sometimes fail due to complications or lack of compliance [8]. Surgical off-loading may then be indicated [9, 10]. The purpose of the operation is to decrease the pressure plantar to the affected metatarsal head. This can be achieved by several surgical techniques including plantar condylectomy, Weil osteotomy with metal fixation, closing wedge metatarsal osteotomy with metal fixation, and Helal osteotomy [11–15]. While all these open methods are effective in off-loading, they tend to have relatively high complication rates because of wound dehiscence and/or post-operative infection. The mini-invasive floating metatarsal osteotomy is an effective off-loading procedure with a low complication rate because soft tissue damage is minimal and metal fixation is not used. The purpose of this retrospective study is to assess the post-operative results of this treatment modality.

## Methods

We performed a retrospective chart review on all patients with DM who had a minimally invasive floating metatarsal osteotomy for the treatment of recalcitrant or recurrent neuropathic plantar metatarsal head ulcers between 2013 and 2014 (inclusion criteria). Patients were identified by ICD codes and operating theater records. All patients were operated on by a single surgeon (ET). No patients were excluded. Parameters collected included demographics, cause of neuropathy, type and duration of DM, recent HbA1c levels, duration and location of current ulcer, location of osteotomy (metatarsal neck or shaft), ulcer status 6 weeks after surgery, and complications. Major complications included any infection necessitating antibiotic treatment. Minor complications were defined as any other adverse event including recurrence, a transfer lesion, and non-union. The study was approved by the Assaf Harofeh Medical Center IRB.

### Operative technique and post-operative management

**Fig. 1:**
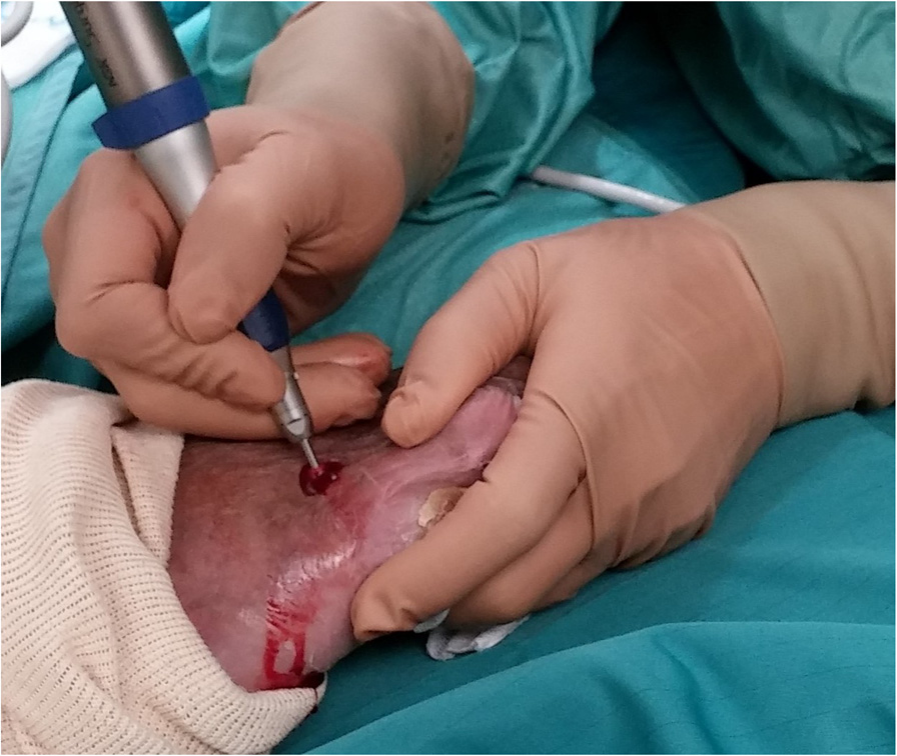
Surgical procedure for metatarsal osteotomy using a 12 × 2 mm Shannon burr

**Fig. 2:**
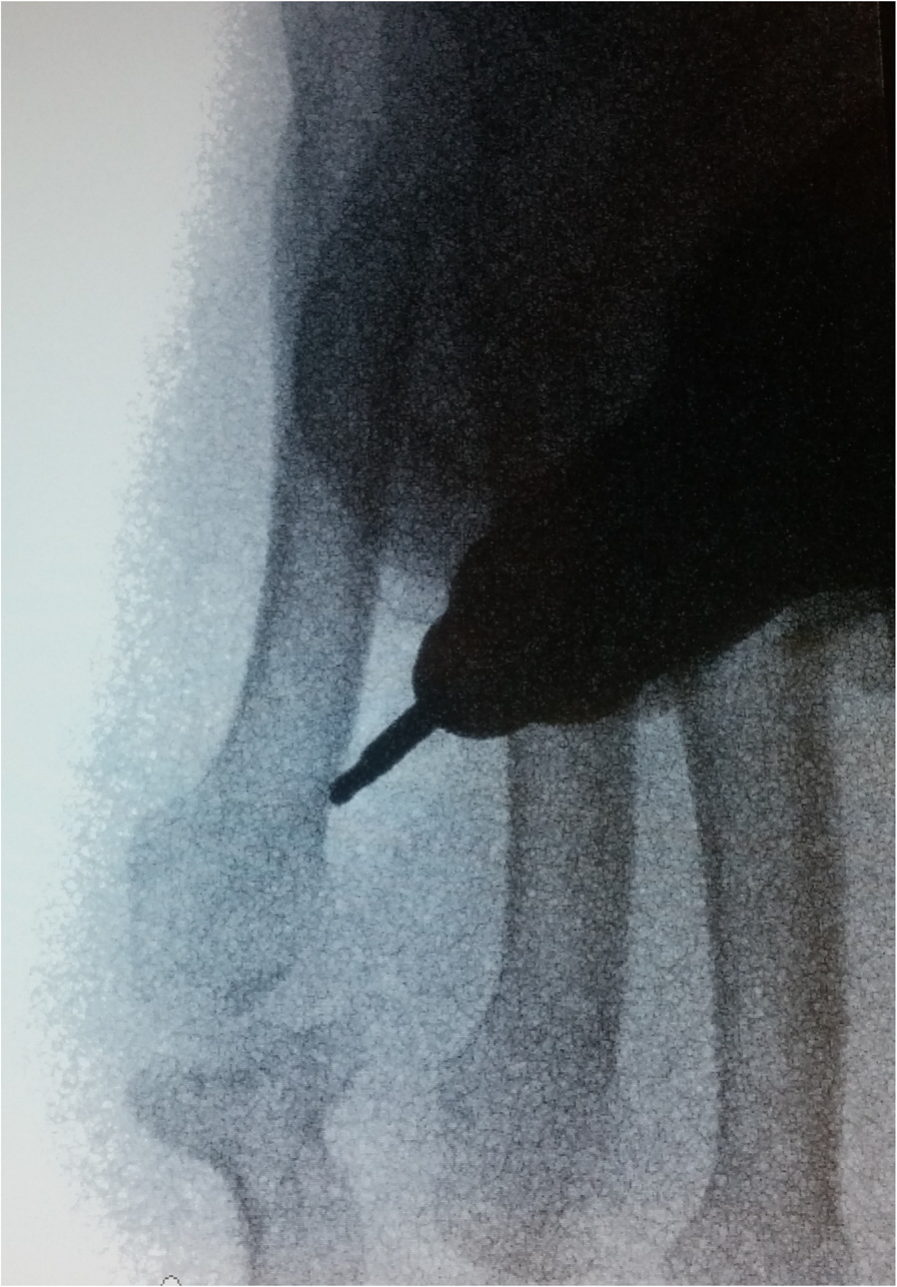
Surgical procedure for metatarsal osteotomy using Shannon burr, fluoroscopic view

**Fig. 3:**
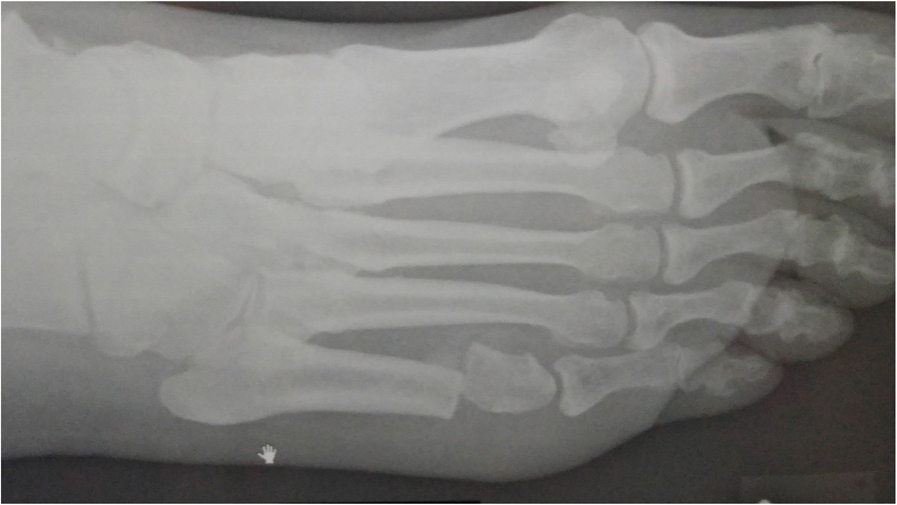
Oblique X-ray of patient in following figures, 1 month following surgery. The fifth MT head is significantly elevated and the osteotomy is healing

Antibiotics (2 gr IV cephalosporin) were given preoperatively. Anesthesia by ankle block was performed with 15 cm^3^ of 1 % lidocaine except in patients with neuropathy severe enough to make anesthesia unnecessary. A 3-mm incision was made dorsally at the planned osteotomy site after fluoroscopic identification. The bone was exposed by blunt dissection with a mosquito. A perpendicular or short oblique osteotomy was made at the neck or diaphysis of the affected metatarsus with a 12 × 2 mm Shannon burr at a speed of 1600 RPM and a torque of 80 Nm (Figs. 1, 2, and 3). Fluoroscopy was used again to confirm completion of the osteotomy. Following the osteotomy, the metatarsal head was displaced dorsally. Skin closure was achieved with a single 4-0 nylon suture. Full weight-bearing in a post-operative shoe was permitted immediately.

## Results

Twenty procedures were performed in 17 patients; all but one were males. Their mean age was 58 (median 57, range 42 to 75, IQR 56 to 63) years. All patients were diagnosed with late-onset DM for a mean and median of 17 (range 4 to 39, IQR 13 to 20) years and their mean HbA1c was 8.1 (median 7.1, range 4.9 to 12.2, IQR 6.5 to 10.5) g/dL. One patient was post-pancreas transplantation. At the time of surgery, 11/17 patients were already being treated with insulin.

All patients had adequate blood supply with palpable pedal pulses or ABI index above 0.7. All ulcers were classified as University of Texas 1A (no infection, no ischemia, ulcer not penetrating to deep structures) [16] and the ulcer’s mean age was 19 (median 11, range 1 to 60, IQR 4 to 36) months. Of 18 primary procedures, in 15 the floating osteotomy included 1 metatarsal and in 3 the osteotomy included 2 metatarsals (the additional metatarsal was always the fourth, secondary to the third). Case details are presented in Table 1.

**Table 1:** Details of osteotomies and results

	Variable	Value (%)
Metatarsal		
	2	5 (25 %)
	3	4 (20 %)
	3 and 4	3 (15 %)
	4	8 (40 %)
Side		
	Right	14 (70 %)
	Left	6 (30 %)
Osteotomy location		
	Neck	12 (60 %)
	Shaft	8 (40 %)
Healed		
	Yes	17 (85 %)
	No	3 (15 %)
Union		
	Yes	14 (70 %)
	No	6 (30 %)
Total		
		20 (100 %)

One patient had a simultaneous bilateral procedure and two patients had second procedures due to the development of transfer ulcers. With the exception of one case, all surgical wounds (95 %) healed within 1 week. In that case, an early post-operative infection with osteomyelitis developed at the osteotomy site (proximal shaft of the fifth metatarsal). The patient was successfully treated by hospital admission with bone debridement and IV antibiotics per culture.

After 17/20 operations, the ulcer completely resolved after 6 weeks (no callus plantar to the affected metatarsal head) and did not recur after a mean follow-up of 11.5 (median 10, range 3 to 21, IQR 8 to 16) months. The remaining three patients showed improvement but were not cured.

Non-union (lack of solid bone formation across the osteotomy site 6 months after the procedure) developed in six cases (30 %), three after osteotomy of the neck and three after osteotomy of the shaft. All patients with non-union were asymptomatic. Two patients developed a transfer lesion, one below the fourth metatarsal head 5 months after an osteotomy of the second metatarsal neck and the other below the second metatarsal head, 10 months after osteotomy of the third metatarsal neck.

## Discussion

Using mini-invasive floating metatarsal osteotomy, we attained an 85 % cure rate in the treatment of University of Texas grade 1A persistent or recurrent foot ulcers. In the long run, these ulcers are known to carry considerable morbidity in patients with DM and have been implicated as a causative factor for infection, hospitalization, amputations, and death.

Surgery correcting foot deformities, thereby decreasing the plantar pressure (internal off-loading), is indicated when conservative methods fail (usually after at least 6 weeks of off-loading with orthotic treatment). This failure might be related to complications of the off-loading, lack of compliance, persistence of pressure below a metatarsal head, or recurrent ulceration despite the off-loading treatment with shoes and orthotics. Contraindications for surgery include ischemia, infection, and ulcers penetrating deep structures.

Surgical options for recurrent or recalcitrant ulcers include Achilles tendon lengthening, [17] metatarsal osteotomies, debridement to metatarsal head resection, and limited amputations [18–20]. There are very few references relating to metatarsal osteotomies for off-loading-resistant and recurrent metatarsal plantar ulcers. Fleischli et al. reported 20 diabetic patients who underwent 22 open dorsiflexion metatarsal base osteotomies with internal fixation for treatment of chronic persistent or recurrent neuropathic forefoot ulcers [21]. Complete ulcer healing was noted in 21 cases (95 %). Complications occurred in 15 cases (68 %) including acute Charcot disease (32 %), deep wound infections (14 %), and transfer lesions (9 %). There were no cases of ulcer recurrence.

Tilo reported on 52 patients who had metatarsal osteotomies without internal fixation for the treatment of chronic neuropathic ulcerations [13]. The most common osteotomy used was the osteoclasis type, where a short (partial) osteotomy was made dorsal to the metatarsal neck, part of the neck was removed with a rongeur and the neck fractured by pushing the head dorsally. No early post-operative complications were noted. Re-ulceration developed in 6 % and transfer ulcers developed in 26.5 % of the cases 17 months following the procedure.

Other types of open osteotomies include the Weil osteotomy and a dorsal closing wedge osteotomy with internal fixation with plate and screws. While these procedures are effective in off-loading the ulcer, the post-operative complication rates of infection and wound dehiscence are relatively high.

**Fig. 4:**
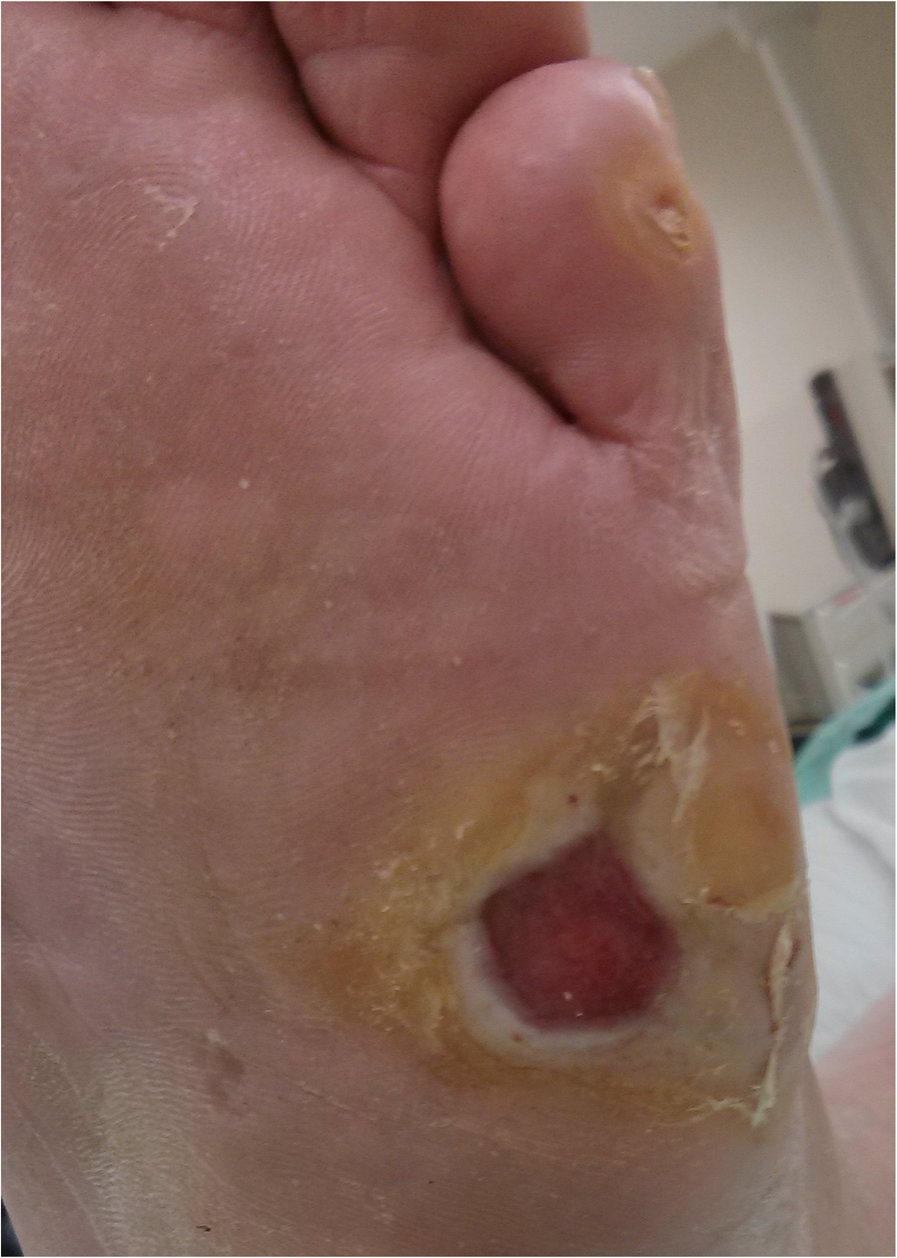
Pre-operative view of chronic recurrent ulcer under the fifth MT head

**Fig. 5:**
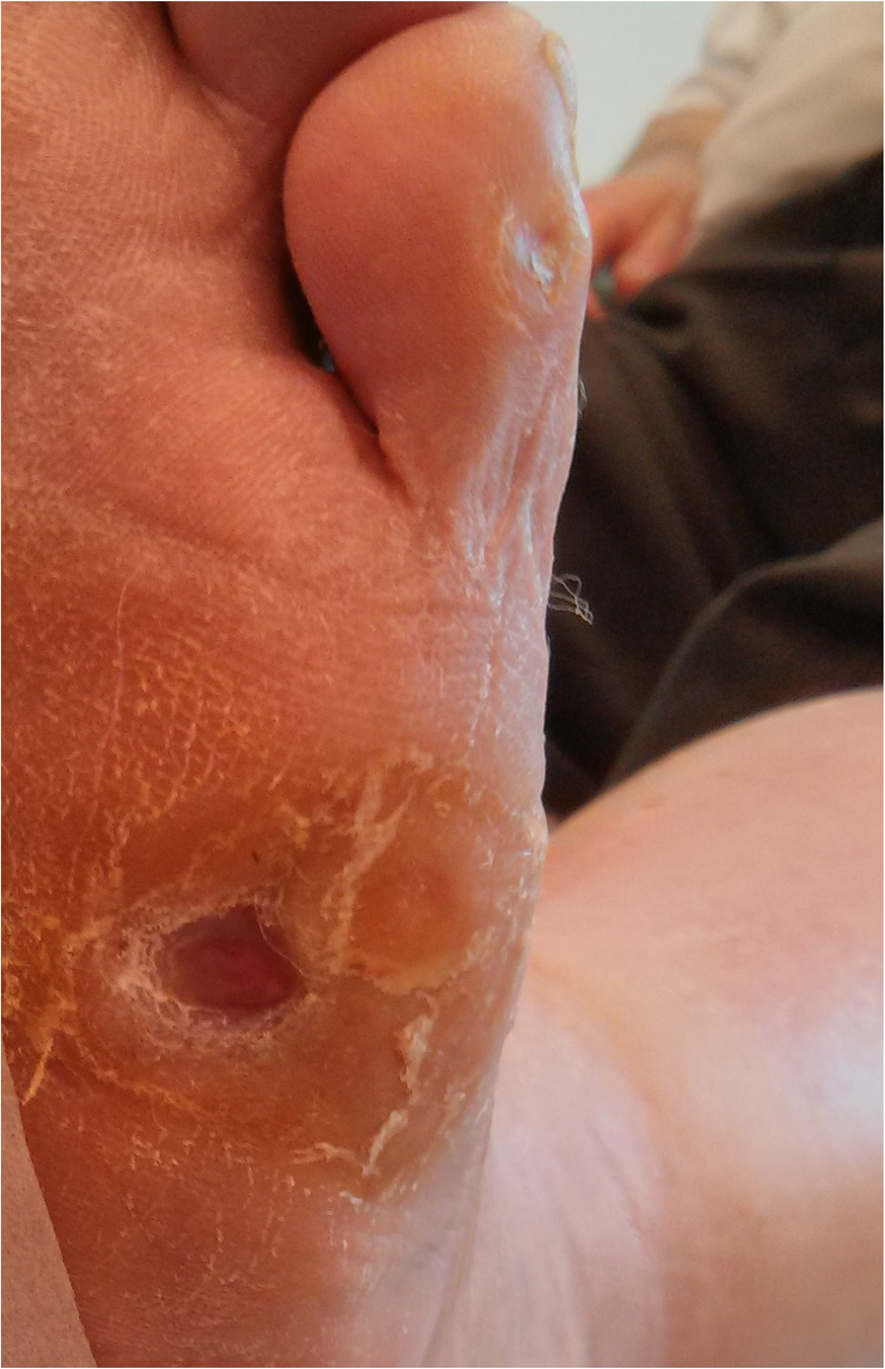
Ulcer, 6 days after surgery. The ulcer is healing rapidly while the patient is fully weight-bearing

**Fig. 6:**
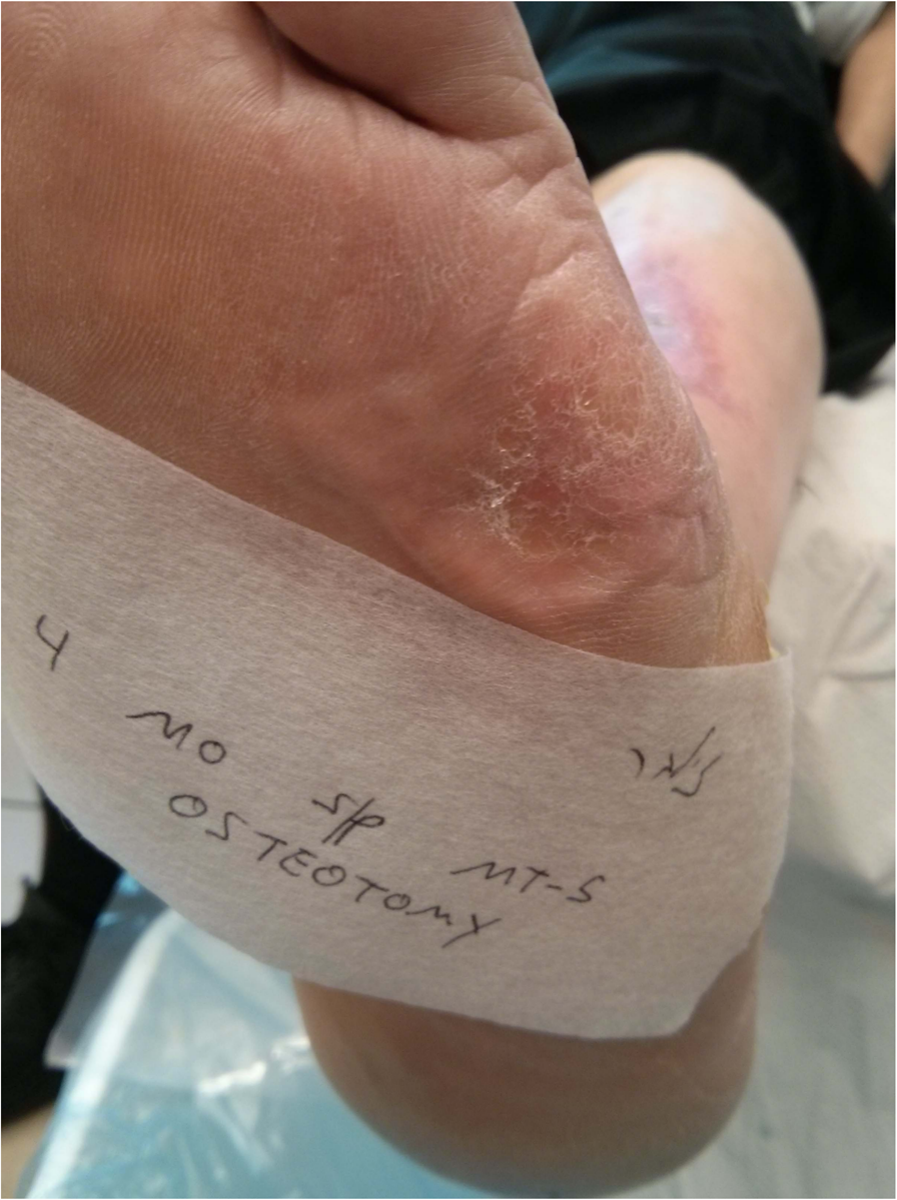
Ulcer site, 4 months after surgery. The skin under the fifth MT head is almost normal

Minimally invasive foot surgery has become quite popular among foot surgeons during the last decade, due to safer methods that use especially designed drills with low speed and high torque to prevent damage to nerves and blood vessels. Applying a minimally invasive technique to metatarsal osteotomies offers effective off-loading with a very low complication rate due to the minimal soft tissue damage. Moreover, the patient is permitted full weight-bearing in a post-operative shoe making the post-operative period easier for the patient. No internal fixation is used (floating osteotomy) and the affected metatarsal head is elevated by the weight-bearing and “settles” in its new position (Fig. 3). The plantar soft tissue reacts rapidly to the off-loading with healing of the ulcer, elimination of the callus, and reappearance of normal skin at the affected area (Figs. 4, 5, and 6).

The non-union rate was quite high (30 %) in this study, both at the neck and the diaphysis, but was asymptomatic in all patients and without clinical sequela. There were no cases of acute Charcot osteo-arthropathy. Two patients developed a transfer lesion 4 and 10 months after the procedure, which were successfully treated with another osteotomy. In three cases, the osteotomy failed to off-load the metatarsal head. One of these was a fourth metatarsal neck osteotomy performed on a foot following an open fifth ray resection, in a morbidly obese patient. Although the post-operative X-ray demonstrated significant elevation of the fourth metatarsal head, the ulcer only decreased in size but did not completely heal after 6 weeks. In the other two cases, the metatarsal head could not be elevated intra-operatively when pushed upwards after a complete osteotomy was done, probably due to fibrosis of the inter-metatarsal ligaments and other soft tissues.

There was one post-operative infection at the osteotomy site. This osteotomy was performed at the proximal diaphysis of a fifth metatarsus for an ulcer under the head in a morbidly obese patient. The patient was not compliant and returned to work 2 days following surgery. He returned 2 weeks later with a purulent discharge from the osteotomy site. He was admitted to hospital for bone debridement and culture-directed antibiotic treatment. Eventually, he recovered with a non-union. On review of this case, we concluded that the reason for this complication was probably soft tissue damage at the osteotomy site caused by the movements of the fifth metatarsal and the long lever arm. Therefore, it is probably safer to perform a fifth metatarsal osteotomy at the neck and not in the diaphysis.

## Conclusions

Mini-invasive floating metatarsal osteotomy is a safe and effective curative and prophylactic procedure for resistant and recurrent University of Texas grade 1 neuropathic plantar metatarsal head ulcers. Teams treating patients with complications of diabetes should include clinicians able to perform advanced curative and prophylactic surgery where appropriate.

## Abbreviations

DM, diabetes mellitus; HbA1c, hemoglobin A1c; ICD, international classification of diseases; IRB, institutional review board; IQR, interquartile range; IV, intravenous; MT, metatarsal; RPM, rounds per minute
